# The upper midwest health study: a case–control study of pesticide applicators and risk of glioma

**DOI:** 10.1186/1476-069X-11-39

**Published:** 2012-06-12

**Authors:** James H Yiin, Avima M Ruder, Patricia A Stewart, Martha A Waters, Tania Carreón, Mary Ann Butler, Geoffrey M Calvert, Karen E Davis-King, Paul A Schulte, Jack S Mandel, Roscoe F Morton, Douglas J Reding, Kenneth D Rosenman

**Affiliations:** 1 National Institute for Occupational Safety and Health, Cincinnati, OH, USA; 2 National Cancer Institute, Rockville, MD, USA; 3 University of Minnesota, Minneapolis, MN, USA; 4 Mercy Foundation, Des Moines, Iowa, USA; 5 Marshfield Clinic, Marshfield, Wisconsin, USA; 6 Michigan State University, East Lansing, MI, USA

**Keywords:** Pesticides, Glioma, Brain cancer, Upper Midwest, Case–control, Farmers, Applicators, Gardens

## Abstract

**Background:**

An excess incidence of brain cancer in farmers has been noted in several studies. The National Institute for Occupational Safety and Health developed the Upper Midwest Health Study (UMHS) as a case–control study of intracranial gliomas and pesticide uses among rural residents. Previous studies of UMHS participants, using “ever-never” exposure to farm pesticides and analyzing men and women separately, found no positive association of farm pesticide exposure and glioma risks. The primary objective was to determine if quantitatively estimated exposure of pesticide applicators was associated with an increased risk of glioma in male and female participants.

**Methods:**

The study included 798 histologically confirmed primary intracranial glioma cases (45 % with proxy respondents) and 1,175 population-based controls, all adult (age 18–80) non-metropolitan residents of Iowa, Michigan, Minnesota, and Wisconsin. The analyses used quantitatively estimated exposure from questionnaire responses evaluated by an experienced industrial hygienist with 25 years of work on farm pesticide analyses. Odds ratios (ORs) and 95 % confidence intervals (CIs) using unconditional logistic regression modeling were calculated adjusting for frequency-matching variables (10-year age group and sex), and for age and education (a surrogate for socioeconomic status). Analyses were separately conducted with or without proxy respondents.

**Results:**

No significant positive associations with glioma were observed with cumulative years or estimated lifetime cumulative exposure of farm pesticide use. There was, a significant inverse association for phenoxy pesticide used on the farm (OR 0.96 per 10 g-years of cumulative exposure, CI 0.93-0.99). No significant findings were observed when proxy respondents were excluded. Non-farm occupational applicators of any pesticide had decreased glioma risk: OR 0.72, CI 0.52-0.99. Similarly, house and garden pesticide applicators had a decreased risk of glioma: OR 0.79, CI 0.66-0.93, with statistically significant inverse associations for use of 2,4-D, arsenates, organophosphates, and phenoxys.

**Conclusions:**

These results are consistent with our previous findings for UMHS of reported farm pesticide exposure and support a lack of positive association between pesticides and glioma.

## Background

Several studies have shown that working on a farm or in the agricultural industry, particularly for men [1,2], may lead to higher risks of glioma and other brain cancer [3-8], while others have not [9-12].

The National Institute for Occupational Safety and Health (NIOSH) developed the Upper Midwest Health Study (UMHS) as a case–control study of intracranial gliomas among rural residents. The primary objective of the UMHS was to determine if pesticides were associated with an increased risk of glioma, the most common type of brain tumor in adults [13]. Gliomas were studied to increase the homogeneity of the case group, in contrast with previous studies that have combined different types of brain tumors with likely different etiologies [14].

We found previously that decreased glioma risks were associated with exposure to farm fumigants among men and women, and to insecticides and organochlorine pesticides among men [15,16]. Modest increased risks were associated with exposure to carbamate fungicides and herbicides and dinitroanilines. These papers used “ever-never” exposure to farm pesticides and analyzed men and women separately. The present paper focuses on pesticide applicators, uses quantitatively estimated exposure on farm pesticide use and includes both men and women in analyses.

The number of farm pesticide applicators is far smaller than the number of individuals living on farms where pesticides are used. Applicators, however, have pesticide exposure levels higher than nonusers [17-20]. This article focuses on the epidemiological evidence in the UMHS between gliomas and farm, other occupational/non-farm, and house and garden pesticide use.

## Methods

The study design and population have been described previously [21]. Briefly, participants were age 18–80 (at ascertainment or diagnosis in 1995 through January 1997) residing in four states (Iowa, Michigan, Minnesota and Wisconsin) in counties where the largest population center had fewer than 250,000 residents. A coordinating center in each state enrolled medical facilities, oncologists, and neurosurgeons, and checked back with all of them periodically for any newly diagnosed cases. In addition, the state cancer registries glioma registrations were reviewed to ascertain any missed diagnoses. Cases with a histologically confirmed primary intracranial glioma (International Classification of Diseases for Oncology [ICD-O] codes 938–948) were identified. Cases with a prior malignancy other than a glioma were not excluded. Physician consent was obtained before contacting cases or their next-of-kin. Controls age 18–64 were randomly selected from state driver’s license/nondriver ID records, and those age 65–80 were selected from Health Care Financing Administration's (HCFA) Medicare data within 10-year age group strata, with the proportion/stratum determined by the age distribution of glioma cases in that state from 1992 to 1994. Controls were frequency-matched within a state but not by county of residence, and were selected even if they had a self-reported history of cancer other than glioma.

After mailing requests for participation, interviewers telephoned to arrange interview appointments. Enclosed with a letter confirming the interview appointment was a list of pesticides used recently and historically in the study states [22]. Participants were asked to report lifetime pesticide use on the farm, at other non-farm jobs, and in the house and garden through 1992, not limited to pesticides on the supplied list. Before the interview the interviewer administered informed consent. Proxy respondents were used when the study participant had died or was too impaired to answer the questionnaire. The questionnaire, modified for use in the present study, was based on one developed by the National Cancer Institute [23].

Participants who applied pesticides (including specific insecticides, herbicides, fungicides, and fumigants/miticides) were asked for first year of use, number of years of use, and days per year of use. Farm insecticide users were also asked whether the insecticide was used on milk cows, other livestock, grains, vegetables, fruit, or farm buildings/lots, and these targets were grouped in three categories in analyses: application to animals (milk cows and other livestock), to crops (grain, vegetable and fruit crops), and to buildings or lots. Since a participant could report having applied multiple pesticides, all reported pesticides were treated individually.

Data were imputed for applicators with missing information such as first year of use, years of use, days per year of use, and targets. Estimated last year of use was derived from first year of use plus the number of years of use. Where the first year of use was missing for a participant, the median age at first application (the difference between first year of use and year of birth) by participants 16 or older (an arbitrary decision for minimum age of application) was calculated (31 years) and added to his or her year of birth. Analyses were conducted using two different exposure estimates described below and on farm pesticides which had been reported as having been applied 100 or more times.

The first exposure set of analyses was for cumulative years of use of each type of pesticide, which was calculated as reported or adjusted years of use for each target multiplied by days per year of use (frequency), divided by 365.25, and then summed across all targets for each participant. Approximately 2 % of all reported pesticide uses had adjusted years of use based on imputed first year of use. Days per year of use were truncated at 250 days per year (n = 4). Zero years of use were assigned to nonusers (n = 1,328) and those with missing days per year of use (8 % of all reported pesticide uses).

The second set of analyses relied on exposure assessment by an experienced industrial hygienist with 25 years of work assessing pesticide exposures (PAStewart). The assessment developed estimates for lifetime cumulative exposure by incorporating estimated intensity levels. The exposure assessor was blinded to the case–control status. Intensity (in mg/hr of dermal exposure levels; the likely substantially lower inhalation level was not estimated) was based on a literature review of over 100 papers reporting pesticides measurements. Over 20 papers identified measurements on animal (1990s) or crop farmers (1960s, 1980s and 1990s) (Appendix A). The measurements covered a variety of pesticides and were reported using a variety of summary statistics, so that while arithmetic averages of the measured exposure levels, weighted for the number of measurements in each study, were calculated, estimates of variability were not. The variability is, however, likely to be high. The mid-points of the averages calculated for crop applications in the 1960s and the 1980s were used as the estimates for the 1970s. The ratio of each decade's average exposure level for crop applications, compared to that for the 1990s, was applied to the applications to animals to derive the 1960s-1980s decennial changes in exposure rates. No data for periods before the 1960s were available for either target, so values for the 1960s were assigned to prior decades. The estimated intensities in the 1960s and prior, the 1970s, the 1980s and the 1990s, respectively, were 113, 78, 43 and 17 mg/hr for applications to animals, and 236, 147, 58, and 22 mg/hr for crop applications. The estimated intensity of insecticide applications to buildings and fences was derived from measurements taken during garden spraying with a handheld sprayer, with no change over time assumed. The estimated intensity for this use was 10 mg/hr. Intensity values for insecticide application to animals and buildings/lots were not applied to herbicide or fungicide exposures because use of these chemicals was assumed to have been on crops only. Hours per day was imputed with the following estimates: 1–2 (i.e., 1.5) hours per day for animals and buildings/lots and 6–8 (i.e., 7) for crops.

In addition to year of first use, several other metrics were imputed for the lifetime cumulative exposure analysis. A probability estimate of applications on animals, crops or buildings was assigned if missing, based on the proportion of non-missing users who applied pesticides to that target. The questionnaire did not enable reporting different numbers of days per year for multiple insecticide targets; therefore for participants with more than one target, the days were proportioned to each target based on the distribution of all single targets. The number of days, if missing, was estimated by decade for herbicides and fungicides from the median of the non-missing reports. Years of use were adjusted based on the EPA registration dates. Cumulative exposure (g-years) for each decade was calculated as the product of intensity (mg/hr), number of hours per day (hr/day), number of days per year (days/yr), and years in that decade, and then summed across all decades for cumulative pesticide exposure. This calculation was done for each type of pesticide, and for insecticides, each target (animals, crops and building/lot) and then summed for each participant to derive lifetime cumulative exposure in grams. Zero years of use were assigned to nonusers.

For both cumulative years and exposure of farm pesticide use, analyses were also conducted using chemical property-based categories and adjusted years based on EPA registration dates. The pesticide list sent to the participants prior to the interview and all pesticide responses were used to develop the NIOSH pesticide reference database [24], a relational data base that associates trade names with the appropriate generic name(s). For example, Bronco, Bullet, Cannon, Freedom, and Lariat are all linked to alachlor. A reported pesticide trade name also could be associated with more than one pesticide, as some trade name pesticides contain more than one pesticide. These pesticides were further categorized into groups based on chemical similarity. If a participant reported using a pesticide before it was on the market, years of use were reduced.

Exposure assessment for nonfarm occupational pesticide and house and garden use was derived directly from the questionnaire responses, and a binary variable of ever/never use of any pesticide was created for analysis.

Unconditional logistic regression modeling adjusted for the frequency-matching variables (10-year age group and sex) and for age and education (a surrogate for socioeconomic status: less than high school, high school graduate, and college graduate (referent group)) was conducted for cumulative years and estimated lifetime cumulative exposure of farm pesticide use. Age was included as well as age group to adjust for residual confounding within age groups [25,26]. Sex was included as men and women differ in pesticide use [27,28]. In addition, stratified analyses by sex were also conducted. One participant with missing education was assigned high school graduate, the most frequently reported education level among participants. Because 98 % of study participants and state residents were white, race was not used as a covariate. Odds ratios and 95 % confidence limits were computed to determine if a risk factor was associated with increased odds for developing intracranial brain gliomas. Rather than make statistical adjustments for the type of respondent, separate analyses were conducted, with risk estimated using data from participants only and from participants and proxies combined. All the analyses used SAS software version 9.2 (SAS Institute Inc. Cary, NC).

This study was approved by the NIOSH Human Subjects Review Board (HSRB 94-DSHEFS-08) and by review boards at every participating institution and was conducted in accordance with subsection (m) of the Privacy Act of 1974 (5 U.S.C. 552a) and Section 308(d) of the Public Health Service Act (42 U.S.C. 242 m) to safeguard individuals and establishments against invasions of privacy.

## Results

There were 228 (29 % of 798) cases and 417 (35 % of 1,175) controls who reported applying pesticides on the farm, for a total of 645 users among 1,973 participants. Table 1 shows the demographic characteristics of all study participants and those who used pesticides on the farm. Compared with all participants, cases and controls who used farm pesticides were, on average, three years older, less likely to have graduated from college, and more likely to be male. Controls were, on average, older than cases (p < 0.05). A total of 4,050 pesticide uses (1,737 insecticide, 2,121 herbicide, 102 fungicide, and 90 fumigant/miticide) were reported. Table 2 shows mean cumulative years of use of pesticide and adjusted glioma odds ratios associated with farm pesticide uses by major type and target, with and without proxy respondents. There was no positive association between cumulative years of use of any farm pesticide on any target and glioma risk. Similarly, no significantly positive findings were observed between glioma risk and estimated lifetime cumulative exposure (Table 3). The numbers of cases and controls who reported having applied pesticides and the numbers having estimated exposures differ because the former were based entirely on questionnaire data with zeros assigned to those with missing values, while the latter were estimated based on literature reviews and expert judgment. The numbers in Table 2 also represent applicators who applied that particular pesticide while those in Table 3 indicate applicators whose exposures could be estimated. The estimated cumulative exposures for a few applicators were zero because a variable used in calculating cumulative exposure was zero. A sensitivity analysis excluded all applicators with zero exposures. While the mean years of use and cumulative exposures increased as expected, the risk estimates were very close (results not shown).

**Table 1:** Characteristics of all participants and farm pesticide applicators in the Upper Midwest Health Study

	**All participants**				**Farm Pesticide Applicators**			
	**Including proxy respondents**		**Excluding proxy respondents**		**Including proxy respondents**		**Excluding proxy respondents**	
**Characteristic**	**Cases**	**Controls**	**Cases**	**Controls**	**Cases**	**Controls**	**Cases**	**Controls**
	**(n = 798)**	**(n = 1,175)**	(n = 438)	(n = 1,141)	(n = 228)	(n = 417)	(n = 125)	(n = 408)
Age^a^ Mean (S.D.)	51.8 (16.1)	54.6 (15.4)	45.4 (15.3)	54.2 (15.4)	54.2 (14.1)	57.3 (13.7)	49.6 (13.5)	57.0 (13.6)
Sex								
Male	457 (57 %)	648 (55 %)	242 (55 %)	625 (55 %)	137 (60 %)	268 (64 %)	75 (60 %)	263 (64 %)
Female	341 (43 %)	527 (45 %)	196 (45 %)	516 (45 %)	91 (40 %)	149 (36 %)	50 (40 %)	145 (36 %)
Race								
White	783 (98 %)	1,152 (98 %)	429 (98 %)	1,119 (98 %)	228 (100 %)	415 (99.5 %)	125 (100 %)	406 (99.5 %)
Non-White	15 (2 %)	23 (2 %)	9 (2 %)	22 (2 %)	0 (0 %)	2 (0.5 %)	0 (0 %)	2 (0.5 %)
State of Residence								
I owa	190 (24 %)	302 (26 %)	101 (23 %)	284 (25 %)	64 (28 %)	121 (29 %)	34 (27 %)	115 (28 %)
Michigan	246 (31 %)	298 (25 %)	133 (30 %)	292 (26 %)	42 (18 %)	75 (18 %)	23 (18 %)	74 (18 %)
Minnesota	163 (20 %)	257 (22 %)	96 (22 %)	252 (22 %)	51 (22 %)	101 (24 %)	29 (23 %)	100 (25 %)
Wisconsin	199 (25 %)	318 (27 %)	108 (25 %)	313 (27 %)	71 (31 %)	120 (29 %)	39 (31 %)	119 (29 %)
Education^b^								
College Graduate	132 (17 %)	200 (17 %)	89 (20 %)	198 (17 %)	32 (14 %)	44 (11 %)	22 (18 %)	44 (11 %)
High School Graduate	523 (66 %)	768 (65 %)	303 (69 %)	752 (66 %)	130 (57 %)	272 (65 %)	82 (66 %)	267 (65 %)
<12 Years	143 (18 %)	207 (18 %)	46 (11 %)	191 (17 %)	66 (29 %)	101 (24 %)	21 (17 %)	97 (24 %)

^**a**^Age on January 1, 1993. Eligibility requirement was age > 18 at time of diagnosis or control selection.

^b^One control with unknown education was assigned with ‘High School Graduate’.

**Table 2:** Risk of glioma and years of use of farm pesticide by type and target among applicators and nonusers in the Upper Midwest Health Study

**Pesticide**	**Including proxy respondents**					**Excluding proxy respondents**				
Type and Target	Cases (n = 798)		Controls (n = 1,175)			Cases (n = 438)		Controls (n = 1,141)		
	n^a^	Years of use^b^	n	Years of use	OR^c^ (95 % CI)	n	Years of use	n	Years of use	OR (95 % CI)
Insecticide use	196	1.20 (3.30)	361	1.26 (2.93)	0.97 (0.92, 1.03)	109	0.96 (2.23)	353	1.27 (2.96)	0.97 (0.89, 1.05)
Animals	77	2.55 (4.69)	166	2.30 (3.83)	0.97 (0.92, 1.03)	44	1.94 (2.90)	163	2.31 (3.86)	0.97 (0.89, 1.06)
Crops	64	0.45 (1.16)	140	0.58 (2.02)	0.88 (0.68, 1.13)	38	0.43 (1.37)	136	0.59 (2.05)	0.94 (0.71, 1.25)
Buildings/Lots	46	1.92 (4.64)	122	1.49 (3.13)	0.97 (0.89, 1.06)	28	1.07 (2.24)	118	1.50 (3.18)	0.94 (0.79, 1.11)
Herbicide use	160	0.26 (0.74)	265	0.41 (0.98)	0.78 (0.59, 1.01)	90	0.25 (0.68)	260	0.42 (0.99)	0.81 (0.58, 1.14)
Fungicide use	29	0.12 (0.28)	45	0.15 (0.38)	0.81 (0.21, 3.17)	19	0.16 (0.33)	45	0.15 (0.38)	1.41 (0.36, 5.56)

^a^Numbers of participants reported any use of farm pesticide, and if applicable, on a target. All other study participants are nonusers.

^b^Mean (and standard deviation) of cumulative years of use. Cumulative years of use is calculated as reported years of use times days/year (maximum truncated at 250 days/year) divided by 365.25 as the exposure variable. It is calculated for all uses in a pesticide/target category by an applicator then summed for the applicator.

^c^Odds ratio (and 95 % confidence intervals) per cumulative year of use, adjusted for matching variables (10-year age group, sex) and for age, and education.

**Table 3:** Risk of glioma and estimated cumulative pesticide exposure by type and target among participants with or without estimated exposures in the Upper Midwest Health Study

**Pesticide**	**Including proxy respondents**					**Excluding proxy respondents**				
Type and Target	Cases (n = 798)		Controls (n = 1,175)			Cases (n = 438)		Controls (n = 1,141)		
	n^a^	Exposure^b^	n	Exposure	OR^c^ (95 % CI)	n	Exposure	n	Exposure	OR (95 % CI)
Insecticide	128	177 (355)	261	155 (263)	0.99 (0.96, 1.02)	73	139 (265)	255	156 (265)	0.99 (0.94, 1.04)
Animal	89	117 (211)	175	118 (179)	0.97 (0.91, 1.03)	48	106 (156)	171	119 (181)	0.98 (0.90, 1.07)
Crops	78	156 (329)	145	135 (228)	1.00 (0.95, 1.04)	42	120 (290)	140	137 (232)	0.99 (0.92, 1.06)
Buildings/Lots	65	1.96 (5.39)	130	1.99 (5.42)	0.99 (0.96, 1.02)	32	1.05 (1.75)	125	2.05 (5.52)	0.95 (0.88, 1.04)
Herbicide	89	149 (255)	163	212 (344)	0.96 (0.91, 1.00)	53	110 (198)	160	214 (347)	0.94 (0.88, 1.01)
Fungicide	17	69.0 (89.5)	32	97.5 (164)	0.90 (0.72, 1.13)	12	76.3 (92.1)	32	97.5 (164)	0.98 (0.78, 1.23)

^a^Number of participants with exposure estimated by an industrial hygienist. All other study participants are assigned zero exposure.

^b^Mean (and standard deviation) of lifetime cumulative exposure in gram-years (g-years) for participants with estimated exposure. Cumulative exposure is calculated as 1,000 times the product of intensity (mg/hr) by decade, number of hours per day (hr/day), number of days per year (days/yr), and years by decade, and then summed across all decades.

^c^Odds ratio (and 95 % confidence intervals) of lifetime cumulative exposure (per 0.5 g for buildings/lots or per 50 g for all others based on median exposure), adjusted for matching variables (10-year age group, sex) and for age, and education.

After linking the pesticide trade names with the NIOSH pesticide reference database, the number of farm pesticide uses increased by 289 to 4,339, because some common trade name pesticides contained more than one pesticide and thus had more than one property category. Organophosphates had the most pesticide-uses at 792, followed by organochlorines at 668. Results are shown in Table 4. Similar to what was observed with major type of pesticide and targets, there was in general no positive association of glioma risk with pesticides. The only statistically significant finding was a decrease in risk with phenoxy uses in analyses with all respondents (adjusted odds ratio (OR) 0.37 per cumulative year of use; 95 % confidence interval (CI) 0.14-0.93); the finding was no longer significant when proxy respondents were excluded.

**Table 4:** Risk of glioma and years of use of farm pesticide by category among applicators and nonusers in the Upper Midwest Health Study

**Pesticide**	**Including proxy respondents**					**Excluding proxy respondents**				
Category	Cases (n = 798)		Controls (n = 1,175)			Cases (n = 438)		Controls (n = 1,141)		
	n^a^	Years of use^b^	n	Years of use	OR^c^ (95 % CI)	n	Years of use	n	Years of use	OR (95 % CI)
Arsenicals	28	0.03 (0.06)	60	0.09 (0.19)	0.01 (0.00, 1.71)	17	0.03 (0.06)	57	0.09 (0.20)	0.07 (0.00, 14.2)
Benzoic Acid	53	0.09 (0.28)	89	0.11 (0.19)	0.62 (0.13, 2.93)	29	0.05 (0.08)	87	0.11 (0.19)	0.06 (0.00, 2.17)
Carbamates	55	0.14 (0.48)	96	0.10 (0.19)	1.21 (0.47, 3.12)	32	0.15 (0.57)	94	0.11 (0.19)	1.38 (0.48, 3.97)
Chloroacetanilides	71	0.11 (0.23)	112	0.13 (0.25)	0.59 (0.18, 1.92)	37	0.12 (0.24)	110	0.13 (0.25)	0.68 (0.17, 2.77)
Dinitroanilines	56	0.04 (0.09)	85	0.06 (0.11)	0.20 (0.01, 5.41)	28	0.05 (0.11)	83	0.06 (0.11)	0.30 (0.01, 15.0)
Organochlorines	110	0.46 (1.22)	217	0.69 (1.81)	0.86 (0.73, 1.01)	55	0.44 (1.15)	213	0.70 (1.83)	0.86 (0.69, 1.08)
Organophosphates	107	0.47 (1.49)	207	0.55 (1.86)	0.94 (0.80, 1.09)	62	0.25 (0.91)	203	0.56 (1.87)	0.82 (0.57, 1.17)
Phenoxys	92	0.10 (0.27)	172	0.19 (0.42)	0.37 (0.14, 0.93)^*^	55	0.09 (0.17)	170	0.19 (0.43)	0.42 (0.13, 1.44)
Triazines	103	0.12 (0.31)	183	0.19 (0.55)	0.54 (0.26, 1.14)	57	0.11 (0.23)	179	0.20 (0.55)	0.51 (0.18, 1.45)

^a^Numbers of users of farm pesticide. All other study participants are nonusers.

^b^Mean (and standard deviation) of cumulative years of use. Cumulative years of use is calculated as reported years of use (adjusted for available year based on EPA record) times days/year (maximum truncated at 250 days/year) divided by 365.25 as the exposure variable. It is calculated for all uses in a class by an applicator then summed for the applicator.

^c^Odds ratio (and 95 % confidence intervals) per cumulative year of use, adjusted for matching variables (10-year age group, sex) and for age, and education

^*^p < 0.05.

In analyses of the estimated lifetime cumulative exposure of chemical property-based categories, the associations were again overall null (Table 5). Phenoxy pesticide use on the farm was again statistically significantly associated with a decreased glioma risk among all participants (OR 0.96 per 10 g-years of estimated lifetime cumulative exposure, CI 0.93-0.99), and the association was no longer significant after excluding proxy respondents.

**Table 5:** Risk of glioma and estimated cumulative pesticide exposure by category among participants with or without estimated exposures in the Upper Midwest Health Study

**Pesticide**	**Including proxy respondents**					**Excluding proxy respondents**				
Category	Cases (n = 798)		Controls (n = 1,175)			Cases (n = 438)		Controls (n = 1,141)		
	n^a^	Exposure^b^	n	Exposure	OR^c^ (95 % CI)	n	Exposure	n	Exposure	OR (95 % CI)
Arsenicals	15	4.86 (6.55)	36	49.5 (80.9)	0.95 (0.90, 1.00)	9	3.08 (2.18)	34	51.1 (82.8)	0.95 (0.86, 1.03)
Benzoic Acid	29	42.6 (72.6)	55	52.9 (73.4)	0.97 (0.92, 1.03)	16	23.6 (27.4)	54	53.8 (73.7)	0.91 (0.80, 1.04)
Carbamates	34	56.7 (125)	65	43.2 (86.9)	1.00 (0.97, 1.04)	20	55.1 (146)	64	43.8 (87.4)	1.00 (0.96, 1.04)
Chloroacetanilides	39	47.7 (72.1)	69	44.1 (60.0)	0.99 (0.94, 1.04)	19	54.8 (82.4)	67	45.4 (60.4)	0.99 (0.93, 1.06)
Dinitroanilines	29	19.1 (28.8)	47	28.8 (58.5)	0.99 (0.98, 1.01)	16	18.1 (32.0)	46	29.5 (59.0)	0.99 (0.98, 1.01)
Organochlorines	63	88.1 (143)	150	83.1 (145)	0.99 (0.97, 1.01)	31	86.4 (153)	146	83.8 (146)	0.99 (0.96, 1.01)
Organophosphates	66	96.5 (204)	145	81.3 (242)	1.00 (0.98, 1.01)	41	75.2 (219)	141	83.5 (245)	1.00 (0.98, 1.02)
Phenoxys	57	47.6 (83.7)	112	101 (170)	0.96 (0.93, 0.99)^*^	37	35.3 (54.3)	111	102 (170)	0.96 (0.92, 1.01)
Triazines	52	59.6 (86.8)	105	91.8 (194)	0.97 (0.95, 1.00)	27	56.3 (82.4)	103	93.5 (196)	0.97 (0.93, 1.01)

^a^Number of participants with exposure estimated by an industrial hygienist. All other study participants are assigned zero exposure.

^b^Mean (and standard deviation) of lifetime cumulative exposure in gram-years (g-years) for participants with estimated exposure. Cumulative exposure is calculated as 1,000 times the product of intensity (mg/hr) by decade, number of hours per day (hr/day), number of days per year (days/yr), and years by decade, and then summed across all decades.

^c^Odds ratio (and 95 % confidence intervals) of lifetime cumulative exposure (per 1 g for arsenical and dinitroaniline, or per 10 g for all others based on median exposure), adjusted for matching variables (10-year age group, sex) and for age, and education.

^*^p < 0.05.

A sensitivity analysis excluded applicators with no estimated years of use of farm pesticide or cumulative pesticide exposure, and the risk estimates were very close to those obtained with all participants included (results not shown).

In stratified analyses by sex, men in general had longer cumulative years of use of farm pesticide and more estimated cumulative pesticide exposure. Their risk estimates were very similar to those with men and women combined. Women had lower exposure and risk estimates, but many results were unreliable, especially by pesticide category, because of extremely small numbers of cases with positive cumulative years of use of farm pesticide or estimated cumulative pesticide exposure (results not shown).

Table 6 presents results for non-farm occupational pesticide use. Ever nonfarm occupational use of any pesticide among all respondents was associated inversely with glioma risk: (OR 0.72, CI 0.52-0.99 and this effect was somewhat strengthened after excluding proxies (OR 0.59, CI 0.39-0.90). No individual pesticide or broader category of pesticides, with or without proxy respondents, was associated with a statistically significant decrease or elevation in glioma risk.

**Table 6:** Risk of glioma and reported pesticide use in non-farm jobs

**Pesticide**		**Including proxy respondents**			**Excluding proxy respondents**		
	CAS number	Cases (n = 798)	Controls (n = 1,175)	OR^a^ (95 % CI)	Cases (n = 438)	Controls (n = 1,141)	OR (95 % CI)
Any pesticide use in non-farm jobs		65	124	0.72 (0.52-0.99)*	34	124	0.59 (0.39-0.90)*
2,4-D	94-75-7	12	32	0.56 (0.28-1.10)	6	32	0.49 (0.20-1.22)
Dicamba	1918-00-9	4	10	0.55 (0.17-1.79)	3	10	0.81 (0.21-3.10)
Glyphosate	1071-83-6	12	19	0.83 (0.39-1.73)	8	19	0.79 (0.33-1.86)
DDT	50-29-3	11	18	0.93 (0.43-1.99)	4	18	0.70 (0.23-2.14)
Diazinon	333-41-5	10	24	0.61 (0.29-1.29)	8	24	0.81 (0.35-1.87)
Malathion	121-75-5	9	18	0.69 (0.30-1.56)	9	18	1.04 (0.45-2.40)
Carbamates		16	35	0.69 (0.38-1.27)	11	35	0.76 (0.37-1.54)
Organochlorines		21	42	0.74 (0.43-1.28)	10	42	0.63 (0.31-1.30)
Organophosphates		24	51	0.65 (0.39-1.07)	16	51	0.69 (0.38-1.25)
Phenoxys		18	34	0.78 (0.43-1.40)	11	34	0.84 (0.41-1.73)

^*a*^Adjusted for age, 10-year age group, education, sex, and farm pesticide exposure (yes/no).

* p < 0.05.

Table 7 presents results for house and garden pesticide use. Similarly to nonfarm occupational pesticide use, house and garden pesticide use was associated with a decreased risk of glioma: OR 0.79, 95 % CI 0.66-0.93, with statistically significant inverse associations for use of 2,4-D, arsenates, organophosphates, and phenoxys. The significant inverse associations remained for any house and garden pesticide use and use of organophosphates at home after proxy respondents were excluded.

**Table 7:** Risk of glioma and reported house and garden pesticide use

Pesticide		**Including proxy respondents**			**Excluding proxy respondents**		
	CAS number	Cases (n = 798)	Controls (n = 1,175)	OR^a^ (95 % CI)	Cases (n = 438)	Controls (n = 1,141)	OR (95 % CI)
Any pesticide use at home		399	666	0.79 (0.66-0.93)*	204	648	0.70 (0.55-0.89)**
2,4-D	94-75-7	66	145	0.64 (0.47-0.88)**	42	143	0.76 (0.51-1.11)
Dicamba	1918-00-9	27	49	0.76 (0.47-1.24)	16	49	0.87 (0.48-1.58)
Glyphosate	1071-83-6	51	76	0.98 (0.67-1.43)	28	75	0.84 (0.52-1.33)
DDT	50-29-3	24	48	0.81 (0.49-1.35)	11	48	0.81 (0.41-1.61)
Diazinon	333-41-5	57	123	0.66 (0.47-0.92)	36	121	0.75 (0.50-1.12)
Malathion	121-75-5	45	84	0.82 (0.56-1.20)	24	84	0.72 (0.44-1.18)
Arsenates		12	36	0.50 (0.26-0.97)*	5	33	0.43 (0.16-1.14)
Botanicals		22	51	0.73 (0.44-1.23)	12	50	0.82 (0.43-1.59)
Carbamates		86	146	0.92 (0.69-1.23)	47	143	0.91 (0.63-1.32)
Inorganics		16	37	0.66 (0.36-1.20)	6	36	0.42 (0.17-1.03)
Organochlorines		65	137	0.73 (0.53-1.00)	33	137	0.69 (0.46-1.05)
Organophosphates		133	247	0.77 (0.20-0.97)*	71	244	0.69 (0.51-0.94)*
Phenoxys		94	184	0.71 (0.54-0.93)*	57	182	0.80 (0.57-1.12)

^*a*^Adjusted for age, 10-year age group, education, sex, and farm pesticide exposure.

* p < 0.05.

**p < 0.01.

## Discussion

We found no positive associations between glioma risk and the application of any pesticide or any chemical property-based category. These results are similar to those found in our study of glioma in women exposed to farm pesticides, the first investigation of glioma risk and pesticides in women [15]. Reports on associations between brain cancer and farm pesticide use in males have not been consistent in cohort and nested case–control studies of farmers and pesticide applicators, ecological studies, and case–control studies of brain cancer [1,2,16]. Studies of pesticide applicator cohorts have reported brain cancer risks ranging from half of those of the general population to three times as great; statistically significant results almost always were restricted to those over age 65 [23,29-43]. Three case–control studies and one ecological study found a positive statistical association between farm pesticide exposure and gliomas [2,44-46]. In nearly all of these studies, the types of pesticides used and estimates of pesticide exposure were not available. Most did not specify tumor type; however, gliomas are the most common tumors and men, the focus of most pesticide studies, have a higher incidence of glioma than do women [47].

A review of the literature on brain cancer and pesticide exposure concluded that “the results of retrospective case–control studies are conflicting” and that the data were insufficient to assume a causal relationship between pesticide exposure and brain cancer [1]. Since that review, four more studies [9-12] did not find a positive association between pesticide exposure and glioma. In contrast, two French studies have found an increased risk of brain cancer in farmers, particularly in vineyards [46,48], possibly because different distributions of pesticide use occur in France compared with the United States. The International Agency for Research on Cancer (IARC) has reported that there is limited or sufficient evidence that certain specific pesticides are carcinogenic in animals, including some carbamates, organochlorines, organophosphates, and triazines. However, none of these has been associated with glioma in animals [49].

Our population-based case–control study of gliomas is the largest to date focusing on non-metropolitan populations. Because our controls were selected based on the distribution of gliomas by age and gender in the years preceding our study period, case–control differences in age distribution were possible, and indeed occurred: control participants were slightly older than case participants (Table 1). This slight difference, however, is unlikely to have impacted the consistent findings of null or decreased risk. The null or decreased associations between use of farm pesticides and reduced glioma risk might be due to a “healthy farm worker” effect [41,50], or to a positive or inverse association between pesticide exposure and another farm-life variable. The significantly inverse association with farm phenoxy use and glioma was not seen when proxies were excluded. One possible explanation is the loss of power due to decreased number of participants in analyses and hence the wider confidence intervals. Underreporting of farm pesticide use by proxies or memory-impaired case participants would have increased the proportion of “non-applicators” among cases. Because controls generally were not impaired and few of them had proxies, having all control applicators but not all case applicators counted would result in lower observed risks.

Our study provided a quantitative investigation of the association between glioma risk and farm pesticide uses, with cumulative years of use based primarily on questionnaire responses and with lifetime cumulative exposure in grams estimated by an expert from measurement data published in the literature. The units and numbers of participants in the analyses of the two exposure measures differed due to the availability of responses or information, but the results were generally in agreement, showing a lack of positive association between glioma risk and pesticides. In addition, the results did not change when we excluded applicators with zero exposures.

In our analysis of farm activities, ruder and colleagues showed that never immediately washing up or changing clothes after applying pesticides was associated with increased glioma risk, which might appear to contradict our findings in the current analysis [51]. However, as stated, the underlying association might have been with imprudent work practices when handling chemicals, not necessarily specifically with pesticides [51].

A limitation of this study is the high proportion (45 %) of proxy interviews (360/798) for case participants (compared to 34/1175 control interviews that were with proxies). Hospital-based rather than physician-based ascertainment might decrease the proportion of proxy interviews. In the hospital-based National Cancer Institute case–control glioma study only 16 % of glioma patients were interviewed by proxy [52], while the Northern California case–control glioma study, which ascertained cases through a cancer registry, had a proxy-interview rate for cases (46 %) similar to ours [53].

The accuracy and completeness of information given by proxy respondents varies by the relationship to the study participant, the gender, race, and age of the proxy, the specific questions asked, and how long the proxy and study participant lived together [54-57]. In a study that recorded pesticide use, widows and wives (35 % of the proxies) reported “having less knowledge of participants’ pesticide use” than did sons (29 %), brothers (11 %), sisters or daughters (10 %), or other male relatives and friends (15 %) [56]. In another study, responses from proxies were highly correlated with those from participant farmers for specific pesticides, but were less accurate in reporting days of use [58]. Proxy responses for cancer cases showed good positive predictive value for the general variable of living or working on a farm, but lower sensitivity when those proxies were asked about specific pesticides or durations of exposure [59]. Johnson et al. (2003) also found proxies interviewed eight years after participant interviews were in excellent agreement with the original respondents for general questions on “ever farmed” or “ever used pesticides”. Agreement declined for major categories of pesticide (insecticide, etc.) and was lowest (50-75 %) for specific pesticides. Proxies both over- and under- reported pesticide use. Leukemia patients were significantly more likely than controls to report having used pesticides; the leukemia odds associated with pesticides, however, decreased and were non-statistically significant when proxy answers were used. In contrast, self-reports showed no case–control difference for 2,4-D or atrazine exposure while proxy reports produced significant 2.6 and 5-fold ORs for cases for 2,4-D and atrazine, respectively [56]. A study of Parkinson’s disease found much higher specificity (>85 %) than sensitivity for proxy responses on agricultural variables including agricultural work, crop and grain farming, and herbicide, insecticide, and fungicide use [60].

Our *a priori* decision to conduct all analyses with and without proxy responses compensated somewhat for the high proportion of proxy responses. It should be borne in mind that case interviews were with participants whose disease could affect recall (whether by differentially recalling possible exposures or forgetting exposures). The results of the analyses with and without proxies were, however, in general similar.

Some pesticide applicators reported a pesticide use that overlapped with another pesticide for the same use. Both pesticides might have been used in the same year or in alternate years. We only asked for total years of use and did not ask participants for last year of use or whether the years of use were consecutive. Therefore, if any participants had nonconsecutive use patterns our analysis may have overestimated cumulative exposure. This would only have affected the disease risks, however, if cases and controls had very different distributions of use patterns.

Of major concern in case–control studies is the validity and reliability of the pesticide exposure assessment. The exposure calculations presented in this paper were mainly based on interview data (subject to differential and non-differential recall by the participants). We tried to minimize recall bias (the tendency among cases to report exposures more accurately than controls or to over-report, thus inflating risk estimates) by not identifying the study hypotheses to participants. Hoar [61] found that reporting of herbicide use by participants and pesticide suppliers was similar for cancer cases and controls. Thus, there may not be differential bias from reporting. Inaccurate recall of pesticide identity could lead to nondifferential bias, which would bias estimates toward the null. To minimize this in reporting of individual pesticides, respondents were given a detailed list of pesticides, including trade names and common names, to review visually. Unfortunately, many house and garden pesticides, and pesticides used on nonfarm jobs, were identified by trade names that were not specific enough to resolve into chemical components [24]. Moreover, many respondents could not supply the pesticide name but only the target pest or crop or animal being protected.

The strengths of our study include the large number of histologically confirmed gliomas, the use of population-based controls, and an exposure assessment procedure that estimated exposure levels by target and decade based on an extensive literature review of published measurements. The population-based design minimizes potential biases associated with hospital-based designs.

## Conclusions

Our results are consistent with our earlier findings for reported pesticide uses and support a lack of positive association between pesticides and glioma.

## Appendix

**Table 8:** References included in the pesticides exposure assessment literature database. The symbols • and ○ indicate that that type of exposure is or is not discussed in the reference

Farm	Farm- related^1^	Non-farm job^2^	House & garden	Reference
•	○	○	○	Abbott IM, Bonsall JL, Chester G. Hart TB, Turnbull GJ: **Worker exposure to a herbicide applied with ground sprayers in the United Kingdom.***Am Ind Hyg Assoc J* 1987, **48:**167**–**175.
○	•	○	○	Archibald BA, Solomon KR, Stephenson GR: **Estimating primicarb exposure to greenhouse workers using video imaging.***Arch Environ Contam Toxicol* 1994, **27:**126**–**129.
○	•	○	○	Archibald BA, Solomon KR, Stephenson GR: **Estimation of pesticide exposure to greenhouse applicators using video imaging and other assessment techniques.***Am Ind Hyg Assoc J* 1995, **56:**226**–**235.
•	○	○	○	Batchelor GS, Walker KC, Elliott JW: **Dinitroorthocresol exposure from apple-thinning sprays.***Arch Ind Hyg* 1956, **13:**593**–**596.
•	○	○	○	Batchelor GS, Walker KC: **Health hazards involved in use of parathion in fruit orchards of north central Washington.***Arch Ind Hyg* 1954, **10:**522**–**529.
•	•	○	○	Brouwer DH, Brouwer EJ, van Hemmen JJ: **Assessment of dermal and inhalation exposure to zineb/maneb in the cultivation of flower bulbs.***Ann Occup Hyg* 1992, **36**:373–384.
○	•	○	○	Brouwer DH, Brouwer R, de Mik G, Maas CL, van Hemmen JJ: **Pesticides in the cultivation of carnations in greenhouses: Part I-Exposure and concomitant health risk.***Am Ind Hyg Assoc J* 1992, **53:**575**–**581.
○	•	○	○	Brouwer R, Marquart H, de Mik G, van Hemmen JJ: **Risk assessment of dermal exposure of greenhouse workers to pesticides after re-entry.***Arch Environ Contam Toxicol* 1992, **23:**273**–**280.
•	○	○	○	Carman GE, Iwata Y, Pappas JL, O'Neal JR, Gunther FA: **Pesticide applicator exposure to insecticides during treatment of citrus trees with oscillating boom and airblast units. ***Arch Environ Contam Toxicol* 1982, **11:**651**–**659.
•	○	○	○	Chester G, Hatfield L, Hart B, Leppert B, Swaine H, Tummon OJ: **Worker exposure to, and absoprtion of, cypermethrin during aerial application of an “ultra low volume” formulation to cotton. ***Arch Environ Contam Toxicol* 1982, **16:**69**–**78.
•	○	○	○	Chester G, Hart TB: **Biological monitoring of a herbicide applied through backpack and vehicle sprayers.***Toxicol Let* 1986, **33:**137**–**149.
•	○	•	○	Comer SW, Staiff DC, Armstrong JF, Wolfe HR: **Exposure of workers to carbaryl.***Bull Environ Contam Toxicol* 1975, **13:**385**–**391.
○	○	•	•	Cowell JE, Lottman CM, Manning MJ: **Assessment of lawn care worker exposure to dithiopyr. ***Arch Environ Contam Toxicol* 1991, **21:**195**–**201.
•	○	○	○	Davis JE, Stevens ER, Staiff DC, Butler LC: **Potential exposure of apple thinners to phosalone.***Bull Environ Contam Toxicol* 1982, **29:**592**–**598.
•	○	○	○	Davis JE, Stevens ER, Staiff DC: **Potential exposure of apple thinners to azinphosmethyl and comparison of two methods for assessment of hand exposure.***Bull Environ Contam Toxicol* 1983, **31:**631**–**638.
○	○	•	•	Davis J, Stevens E, Staiff D, Butler C: **Potential exposure to diazinon during yard applications.***Environ Mon Ass* 1983, **3:**23**–**29.
•	○	○	○	Devine JM, Kinoshita GB, Peterson RP, Picard GL: **Farm worker exposure to terbufos [phosphorodithioic acid, S-(tert-butylthio) methyl O,O-diethyl ester] during planting operations of corn.***Arch Environ Contam Toxicol* 1996, **15:**113**–**119.
•	○	○	○	Dubelman S, Lauer R, Arras DD, Adams SA: **Operator exposure measurements during application of the herbicide diallate.***J Agric Food Chem* 1982, **30:**528**–**532.
•	○	○	•	Everhart LP, Holt RF: **Potential benlate fungicide exposure during mixer/loader operations, crop harvest, and home use.***J Agric Food Chem* 1982, **30:**222**–**227.
○	•	○	○	Frank R, Campbell RA, Sirons GJ: **Forestry workers involved in aerial application of 2,4-dichlorophenoxyacetic acid (2,4-D): exposure and urinary excretion.***Arch Environ Contam Toxicol* 1985, **14:**427**–**435.
○	•	○	○	Franklin CA, Grover R, Markham JW, Smith AE, Yoshida K: **Effect of various factors on exposure of workers involved in the aerial application of herbicides.***Am Conf Gov Ind Hyg Transact* 1981, **43:**97**–**117.
○	○	•	•	Gold RE, Holcslaw T, Tupy D, Ballard JB: **Dermal and respiratory exposure to applicators and occupants of residences treated with dichlorvos (DDVP).***J Econ Entomol* 1984, **77:**430**–**436.
•	○	○	○	Grover R, Cessna AJ, Nuir NI, Riedel D, Franklin CA, Yoshida K: **Factors affecting the exposure of ground-rig applicators to 2,4-D dimethylamine salt.***Arch Environ Contam Toxicol* 1986, **15:**677**–**686.
•	○	○	○	Herman ND, Hunt TW, Sheets TJ: **Hand harvester exposure to maleic hydroxide (MH) in flue-cured tobacco.***Bull Environ Contam Toxicol* 1985, **34:**469**–**475.
•	○	○	○	Hussain M, Yoshida K, Atiemo M, Johnston D: **Occupational exposure of grain farmers to carbofuran.***Arch Environ Contam Toxicol* 1990, **19:**197**–**204.
•	○	○	○	Jegier Z: **Health hazards in insecticide spraying of crops.***Arch Environ Health* 1964, **8:**670**–**674.
•	○	•	○	Jegier Z: **Exposure to guthion during spraying and formulating.***Arch Environ Health* 1964, **8:**565**–**569.
○	○	•	•	Kamble ST, Ogg CL, Gold RE, Vance AD: **Exposure of applicators and residents to chlordane and heptachlor when used for subterranean termite controls.***Arch Environ Contam Toxicol* 1992, **22:**253**–**259.
○	•	○	○	Kangas J, Laitinen S, Jauhiainen A, Savolainen K: **Exposure of sprayers and plant harvesters to mevinphos in Finnish greenhouses.***Am Ind Hyg Assoc J* 1993, **54:**150**–**157.
•	○	○	○	Karr C, Demers P, Costa LG, Daniell WE, Barnhart S, Miller M, Gallagher G, Horstman SW, Eaton D, Rosenstock L: **Organophosphate pesticide exposure in a group of Washington State orchard applicators.***Environ Res* 1992, **59:**229**–**237.
•	○	○	○	Knarr RD, Cooper GL, Brian EA, Kleinschmidt MG, Graham DG: **Worker exposure during aerial application of a liquid and a granular formulation of Ordram® Selective Herbicide to rice.***Arch Environ Contam Toxicol* 1985, **14:**523**–**527.
•	○	○	○	Kurtz PH, Shaw G, Kelter A, Jackson RJ: **Assessment of potential acute health effects in agricultural workers exposed during the application of chlordimeform.***J Occup Med* 1987, **29:**592**–**595.
○	•	○	○	Lavy TL, Cowell JE, Steinmetz JF, Massey JH: **Conifer seedling nursery worker exposure to glyphosate. ***Arch Environ Contam Toxicol* 1992, **22:**6**–**13.
○	•	○	○	Lavy TL, Shepard JS, Mattice JD: **Exposure measurements of applicators spraying (2,4,5-trichlorophenoxy)acetic acid in the forest.***J Agric Food Chem* 1980, **28:**626**–**630.
○	○	•	○	Leavitt JR, Gold RD, Holcslaw T, Tupy D: **Exposure of professional pesticide applicators to carbaryl. ***Arch Environ Contam Toxicol* 1982, **11:**57**–**62.
•	○	○	○	Maitlen JC, Sell CR, McDonough LM, Fertig SN: **Workers in the agricultural environment: dermal exposure to carbaryl.** In *Pesticide Residues and Exposure. American Chemical Society Symposium Series 182.* Edited by Plimmer JR. Washington DC: American Chemical Society; 1982:83–103.
•	○	○	○	Manninen A, Kangas J, Klen T, Savolainen H: **Exposure of Finnish farm workers to phenoxy acid herbicides.***Arch Environ Contam Toxicol* 1986, **15:**107**–**111.
•	○	○	○	McCurdy SA, Hansen ME, Weisskopf CP, Lopez RL, Schneider F, Spencer J, Sanborn JR, Krieger RI, Wilson BW, Goldsmith DF, Schenker MB: **Assessment of azinphosmethyl exposure in California peach harvest workers.***Arch Environ Health* 1994, **49:**289**–**296.
○	•	○	○	Methner MM, Fenske RA: **Pesticide exposure during greenhouse applications, Part 1. Dermal exposure reduction due to directional ventilation and work training.***Appl Occup Environ Hyg* 1994, **9:**560**–**566.
○	•	○	○	Methner MM, Fenske RA: **Pesticide exposure during greenhouse applications. III. Variable exposure due to ventilation conditions and spray pressure.***Appl Occup Environ Hyg* 1996, **11:**174**–**180.
•	○	•	•	Mumma RO, Brandes GA, Gordon CF: **Exposure of applicators and mixer-loaders during the application of mancozeb by airplanes, airblast sprayers and compressed-air backpack sprayers.** In *Dermal Exposure Related to Pesticide Use. American Chemical Society Symposium Series 273.* Edited by Honeycutt RC, Zweig D, Ragsdale N. Washington DC: American Chemical Society; 1985:201–219.
•	○	○	○	Munn S, Keefe TJ, Savage EP: **A comparative study of pesticide exposures in adults and youth migrant field workers.***Arch Environ Health* 1985, **40:**215**–**220.
○	○	•	○	Nigg HN, Stamper JH: **Exposure of Florida airboat aquatic weed applicators to 2,4-dichlorophenoxyacetic acid (2,4-D).***Chemosphere* 1983, **12:**209**–**215.
•	○	○	○	Nigg HN, Stamper JH: **Exposure of spray applicators and mixer-loaders to chlorobenzilate miticide in Florida citrus groves. ***Arch Environ Contam Toxicol* 1983, **12:**477**–**482.
•	○	○	○	Nigg HN, Stamper JH, Mahon WD: **Handgun applicator exposure to ethion in Florida citrus.***Bull Environ Contam Toxicol* 1990, **45:**463**–**468.
•	○	○	○	Nigg HN, Stamper JH, Queen RM: **Dicofol exposure to Florida citrus applicators: effects of protective clothing. ***Arch Environ Contam Toxicol* 1986, **15:**121**–**134.
○	○	•	○	Ogg CL, Gold RE: **Exposure and field evaluation of fenoxycarb for German cockroach (Orthoptera: Blattellidae) control.***J Econ Entomol* 1988, **81:**1408**–**1413.
•	○	○	○	Popendorf WJ, Spear RC, Leffingwell JT, Yager J, Kahn E: **Harvester exposure to Zolone® (phosalone) residues in peach orchards.***J Occup Med* 1979, **21:**189**–**194.
•	○	○	○	Putnam AR, Willis MD, Binning LK, Boldt PF: **Exposure of pesticide applicators to nitrofen: Influence of formulation, handling systems and protective garments.***J Agric Food Chem* 1983, **31:**645**–**650.
•	○	•	○	Quinby GE, Walker KC, Durham WF: **Public health hazards involved in the use of organic phosphorous insecticide in cotton culture in the delta area of Mississippi.***J Econ Entomol* 1958, **51:**831**–**838.
○	○	•	○	Ringenburg V: *Industrial hygiene characterization of pesticide exposures of Hamilton County park district workers IWS 80-20/HHE 87–241.* Cincinnati OH: NIOSH; 1988.
○	○	•	○	Sanderson WT, Ringenburg V, Biagini R: **Exposure of commercial pesticide applicators to the herbicide alachlor.***Am Ind Hyg Assoc J* 1995, **56:**890**–**897.
•	○	○	○	Senior PL, Lavers A: **Determination of potential dermal exposure during application of crop protection products by boom spraying.***Ann Occup Hyg* 1992, **36:**589**–**599.
•	○	○	○	Spear RC, Popendorf WJ, Leffingwell JT, Milby TH, Davies JE, Spencer WF: **Fieldworkers' response to weathered residues of parathion.***J Occup Med* 1977, **19:**406**–**410.
○	•	○	○	Stamper JH, Nigg HN, Mahon WD, Nielsen AP, Royer MD: **Applicator exposure to fluvalinate, chlorpyrifos, captan, cand chlorothalonil in Florida ornamentals.***J Agric Food Chem* 1989, **37:**240**–**244.
○	•	○	○	Stamper JH, Nigg HN, Mahon WD, Nielsen AP, Royer MD: **Pesticide exposure to a greenhouse drencher.***Bull Environ Contam Toxicol* 1989, **42:**209**–**217.
○	•	○	○	Stamper JH, Nigg HN, Mahon WD, Nielsen AP, Royer MD: **Pesticide exposure to greenhouse handgunners. ***Arch Environ Contam Toxicol* 1989, **18:**515**–**529.
•	○	○	○	Van Hemmen JJ: **Agricultural pesticides exposure data bases for risk assessment.***Rev Environ Contam Toxicol* 1992, **126:**1**–**85.
•	○	○	○	Wojeck GA, Nigg HN, Braman RS, Stamper JH, Rouseff RL: **Worker exposure to arsenic in Florida grapefruit spray operations. ***Arch Environ Contam Toxicol* 1982, **11:**661**–**667.
•	○	○	○	Wojeck GA, Nigg HN, Stamper JH, Bradway DE: **Worker exposure to ethion in Florida citrus. ***Arch Environ Contam Toxicol* 1981, **10:**725**–**735.
•	○	•	○	Wojeck GA, Price JF, Nigg HN, Stamper JH: **Worker exposure to paraquat and diquat.***Arch Environ Contam Toxicol* 1983, **12:**65**–**70.
•	○	•	○	Wolfe HR, Armstrong JF, Staiff DC, Comer SW: **Exposure of spraymen to pesticides.***Arch Environ Health* 1972, **25:**29**–**31.
•	○	○	○	Wolfe HR, Durham WF, Armstrong JF: **Exposure of workers to pesticides.***Arch Environ Health* 1967, **14:**622**–**633.
•	○	○	○	Wolfe HR, Durham WF, Armstrong JF: **Health hazards of the pesticides endrin and dieldrin. Hazards in some agricultural uses in the Pacific Northwest.***Arch Environ Health* 1963, **6:**458**–**464.
•	○	○	○	Wolfe HR, Durham WF, Batchelor GS: **Health hazards of some dinitro compounds. Effects associated with agricultural usage in Washington State.***Arch Environ Health* 1961, **3:**468**–**475.
○	○	•	○	Wolfe HR, Walker KC, Elliott JW, Durham WF: **Evaluation of the health hazards involved in house-spraying with DDT.***Bull World Health Organ* 1959, **20:**1**–**14.
•	○	○	○	Zweig G, Gao R, Popendorf W: **Simultaneous dermal exposure to captan and benomyl by strawberry harvesters.***J Agric Food Chem* 1983, **31:**1109**–**1113.
•	○	○	○	Zweig G, Leffingwell JT, Popendorf W: **The relationship between dermal pesticide exposure by fruit harvesters and dislodgeable foliar residues.***Environ Sci Health B* 1985, **20:**27**–**59.

1.

2. Nonfarm-related pesticide application and pesticide manufacturing workers.

## Abbreviations

CI, 95 % Confidence Interval; DDT, Dichloro-Diphenyl-Trichloroethane; NIOSH, National Institute for Occupational Safety and Health; OR, Adjusted Odds Ratio; UMHS, Upper Midwest Health Study.

## Competing interests

The authors declare that they have no competing interests.

## Authors’ contributions

JHY and AMR performed the statistical analyses, interpreted the results and drafted the manuscript. PAStewart and MAW conducted the quantitative exposure assessment and helped to draft the manuscript. TC reviewed and revised the draft manuscript. AMR, MAB, GMC, KED, PASchulte, JSM, RFM, DJR and KDR designed the study; GMC, KED, AMR, and MAB oversaw the field work; JSM, RFM, DJR and KDR conducted the field work.
